# Supplementary material for: Bioactivity and Sensory Properties of Probiotic Yogurt Fortified with Apple Pomace Flour
Source: Foods. 2020 Jun 10;9(6):763. doi: 10.3390/foods9060763 (PMC7353605; doi:10.3390/foods9060763)
Supplement: Supplementary file 1 [file foods-09-00763-s001.pdf]

Process:	Jabuka			Page:	1 / 1
Charge No:	SF 750 Plus	Company:	IPLB	Tester:	2
Device Name:		Serial Number:	B8170132	Signature:	
Time Range:	20.09.2019 14:01 – 20.09.2019 15:44				

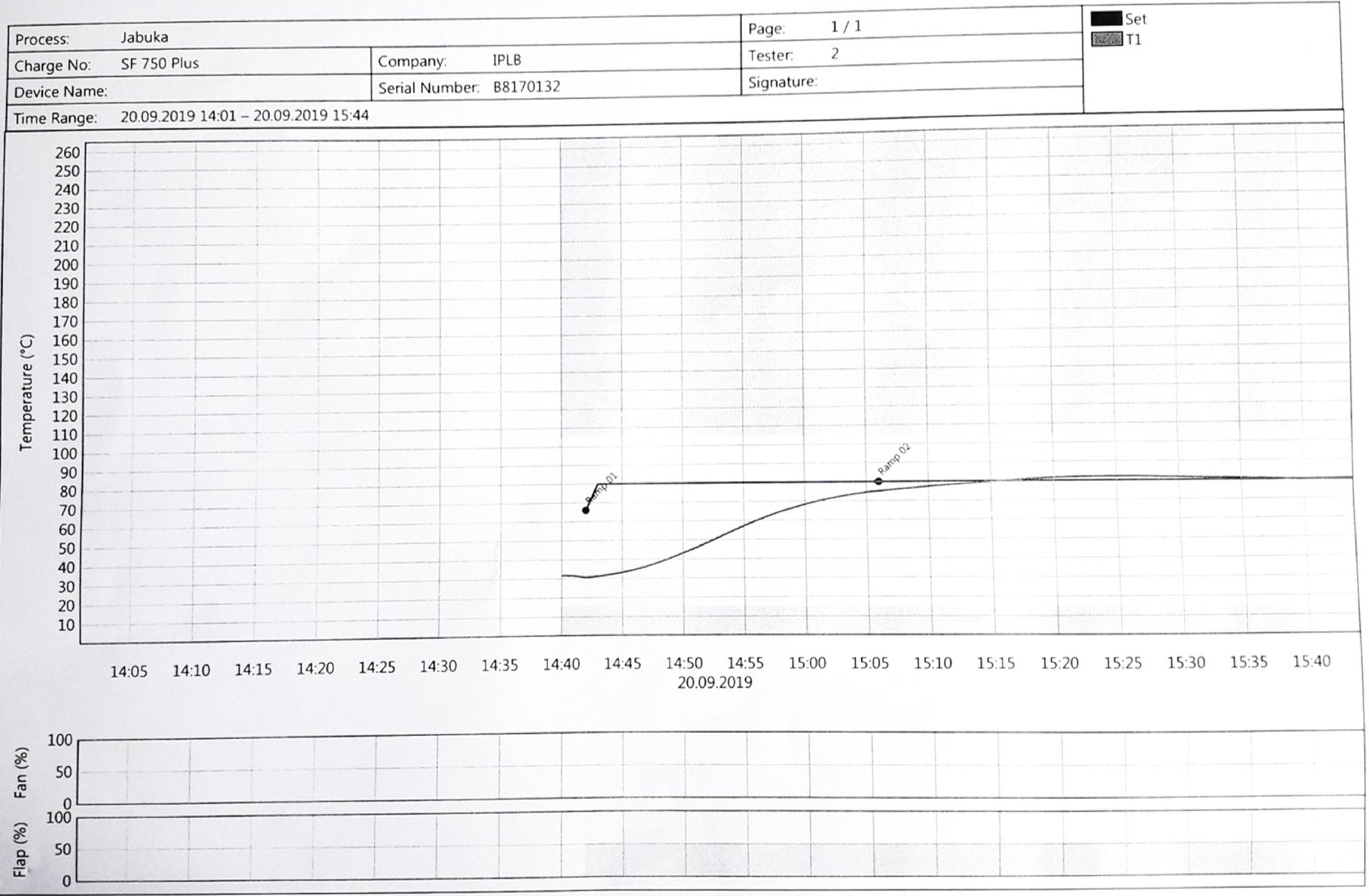

AtmoCONTROL © 2012 - 2015 Memmert GmbH + Co. KG
